# Plasma Exosomal miRNA-122-5p and miR-300-3p as Potential Markers for Transient Ischaemic Attack in Rats

**DOI:** 10.3389/fnagi.2018.00024

**Published:** 2018-02-06

**Authors:** Dong-Bin Li, Jing-Li Liu, Wei Wang, Xiu-Mei Luo, Xia Zhou, Jin-Pin Li, Xiao-Li Cao, Xiao-Hong Long, Jia-Gui Chen, Chao Qin

**Affiliations:** ^1^Department of Neurology, The First Affiliated Hospital, Guangxi Medical University, Nanning, China; ^2^Public Health School of Guangxi Medical University, Nanning, China

**Keywords:** transient ischaemic attack, plasma exosomes, biomarker, rno-miR-122-5p, rno-miR-300-3p

## Abstract

**Background:** Differentiation of transient ischaemic attack (TIA) from ischaemic stroke within the thrombolysis time window is difficult. Although TIA may be diagnosed within this window, the latest imaging technologies are complex and costly. Serum markers, which are non-invasive, rapid and economic, are used for diagnosis and prognosis of various diseases. Exosome-derived miRNA markers for TIA are unknown.

**Methods:** We examined focal brain ischaemia produced by occlusion of the middle cerebral artery (MCAo) for 5 min, 10 min, and 2 h in rats. Exosomal miRNAs with consistent trends in cerebrospinal fluid (CSF) and plasma were identified by deep sequencing and quantitative real-time polymerase chain reaction (qRT-PCR). The areas under the curve (AUC) of the receiver operating characteristic (ROC) curve were used to evaluate the diagnostic accuracy of these miRNAs for TIA in rats.

**Results:** Rno-miR-122-5p and rno-miR-300-3p were selected. Plasma exosomal rno-miR-122-5p was significantly downregulated in 10 min ischaemic rats compared with control and 5 min plasma. Plasma exosomal rno-miR-300-3p was significantly upregulated in 5 min ischaemic rats compared with control, 10 min and 2 h rats. Plasma and CSF levels of these miRNAs were correlated. ROC analysis showed high AUC values for rno-miR-122-5p (0.960) and rno-miR-300-3p (0.970) in the 10 and 5 min rats, respectively, compared with controls.

**Conclusions:** Plasma exosomal rno-miR-122-5p and rno-miR-300-3p may be blood-based TIA biomarkers.

## Introduction

Ischaemic stroke (IS) is a major cause of death and disability worldwide and an important threat to patient health and quality of life (Johnston et al., 2009). It is difficult to differentiate transient ischaemic attack (TIA) from IS within the time window of thrombolysis. Although TIA may be diagnosed within a restricted time window utilizing the latest imaging technologies, the complexity and high cost of these methods limit their widespread use. Multiple blood-based markers are applied for the diagnosis and prognosis of many diseases because these strategies are non-invasive, rapid and economic. Various proteins have been reported to play an important role as blood markers in TIA, which is correlated with oxidative stress, inflammation, neurological damage and vascular endothelial dysfunction (Jickling and Sharp, 2011). However, recent studies have suggested that the predictive value of these proteins is limited (Greisenegger et al., 2015).

MiRNAs are noncoding 20–24-nucleotide-long RNA molecules involved in the post-transcriptional regulation of gene expression by binding to target messenger RNAs (mRNAs). They play an important role in the function and survival of normal neuronal (Singer et al., 2005; Tan et al., 2013). Some miRNAs have been shown to be important in diseases; for example, circulating miRNA-21 is a potential biomarker for IS in humans (Tsai et al., 2013), and miRNA-193b regulates amyloid precursor protein in Alzheimer's disease (Liu et al., 2014). More importantly, study has reported brain-enriched miRNAs play a significant role in the pathophysiological mechanism of IS (Christensen and Schratt, 2018). Research has confirmed that miRNAs in the blood are primarily derived from exosomes, which are small membrane vesicles with a diameter of 30–200 nm that are released as a result of the fusion of multivesicular bodies with the plasma membrane (Balusu et al., 2016; Wang et al., 2017). MiRNAs are particularly abundant in exosomes, which transport miRNAs. Importantly, the exosomal miRNA expression levels can reflect the physiology, pathology and function of cells (Ajit, 2012). Furthermore, compared with miRNAs found directly in the blood, exosomal miRNAs are better protected from degradation, making them more useful biomarkers.

In addition, several studies have reported cerebral spinal fluid (CSF) markers as putative biomarkers for diagnosing diseases. For example, CSF exosomal miR-193b was proposed as a biomarker for Alzheimer's disease (Liu et al., 2014), CSF exosomal miR-15b and miR-21 as markers for gliomas (Baraniskin et al., 2012) and some miRNAs in CSF as biomarkers of stroke (Sørensen et al., 2014). CSF is in direct contact with the brain and reflects the biochemical changes occurring in the brain, but this fluid is obtained by lumbar punctures, which is an invasive and difficult procedure, especially in the elderly. Therefore, we aimed to identify new biological biomarkers that are less intrusive and easily obtained with a high sensitivity and specificity; thus, blood biomarkers would be a good choice. Based on previous studies showing that CSF miRNAs were transported through the blood-brain barrier (BBB) into the bloodstream (Cheng et al., 2013) and that the miRNA CSF profile parallels that of blood, changes occurring in the periphery reflect changes ongoing in the brain, and thus, miRNAs could be useful as non-invasive biomarkers (Galimberti et al., 2014). Studies suggest that the 5 and 10 min middle cerebral artery occlusion (MCAo) model of rats could simulate human TIAs (Zhan et al., 2010), and no permanent neurological deficits observed by laser doppler flowmetry (LDF) or magnetic resonance imaging (MRI) lesions were found in rats with MCAo ≤ 10 min (Pedrono et al., 2010). However, routine haematoxylin and eosin (H&E) and terminal deoxynucleotidyl transferase-mediated dUTP-biotin nick end-labeling (TUNEL) staining indicated that minute ischaemic changes occurred even after 2.5-min MCAo and that some specific gene expression patterns were observed in the blood of rats after TIA (5 and 10 min) compared with IS (2 h) and sham controls (Pedrono et al., 2010; Zhan et al., 2010). In this study, we investigated the expression of miRNAs in CSF and blood of rats with TIA, screened exosome-derived miRNAs that showed a consistent change in the CSF and plasma, and discussed their value as potential biomarkers. This study will contribute to the rapid and efficient diagnosis of TIA, reducing the cost and promoting thrombolysis in acute IS. To the best of our knowledge, this is the first study showing miRNA expression changes in the CSF of rats with TIA.

## Materials and methods

### Animals

Male Sprague–Dawley rats (250 ± 30 g) were used in this study. This study was carried out in accordance with the recommendations of the Council for International Organization of Medical Sciences on Animal Experimentation (World Health Organization, Geneva, Switzerland). The protocol was approved by the Animal Care and Use Committee of the Guangxi Medical University.

In this study, 15 rats were randomly selected and divided into 3 groups according to the occlusive time (5 min, 10 min, and 2 h, *n* = 5 for each group), and 5 rats assigned to the sham group underwent the same surgical protocol but without suture inserted into the external carotid artery. Rat plasma samples were collected for the next experiments. As the CSF samples of rats (approximately 100 μl per rat) had a low volume, we used 5 pooled samples from 15 individual rats for each ischaemia group; in total, 60 rat CSF samples were used.

### MCAo model

MCAo was induced as previously described (Zhao et al., 2010). Briefly, rats were intraperitoneally anesthetized with 3.5% chloral hydrate (1.0 ml/100 g). The right common carotid artery, right external carotid artery and right internal carotid artery were exposed through a short incision. Internal carotid artery was clamped using a bulldog clamp temporarily. Then, monofilament nylon sutures with a round tip (L3600, Jialing, China) were placed into the ECA and advanced into the ICA approximately 18 mm. The sham group underwent the same procedures but without suture insertion. Rats were subjected to 5 min, 10 min, and 2 h of MCAo, and the suture was then removed. Brain tissue, blood and CSF were obtained immediately for the next step.

### Collection of CSF and plasma

CSF (100 μl) was aspirated with a 100 μl microliter syringe (Gaoge, China) from the cisterna magna after careful dural puncture and then transferred to Eppendorf tubes and stored at −80°C until use. Blood samples were drawn immediately after the CSF collection. Then, 5 ml blood samples were obtained from the abdominal aorta, stored in tubes with EDTA anticoagulant, and centrifuged at 3,000 g for 15 min at 4°C. The upper aqueous phase (plasma) was then transferred to Eppendorf tubes and stored at −80°C until use.

### TUNEL assay

After intracardiac paraformaldehyde perfusion, approximately 2 mm brain tissues of rats were obtained from two points, anterior and posterior to the optic chiasm. Brain specimens were immersed in 4% paraformaldehyde individually, fixatived for 24 h and embedded in paraffin for the slicing of 3-μm sections. TUNEL analysis was performed according to the instructions of the TUNEL-POD kit (Roche Applied Science, Germany). Positively labeled nuclei (brown color) were identified from unstained nuclei (blue color). Four visual fields were randomly seleted under a high magnification microscope (100×) were chosen in the frontoparietal cortex of the ischaemic hemisphere. The apoptotic index was calculated based on the percentage of positive apoptotic cells.

### Haematoxylin-eosin staining

Rats were perfusion-fixed with 4% paraformaldehyde in 0.1 M phosphate buffer (pH 7.4) under anesthesia. The paraffin-embedded brain sections were prepared and stained with haematoxylin and eosin. For observation of cell morphology, the pathological sections were observed under a high magnification microscope at 100 × amplification. To assess neuronal damage, we evaluated the presence of shrunken neurons, hypereosinophilic neurons, vacuolization, and loss of affinity for haematoxylin in the frontoparietal cortex of the ischaemic hemisphere.

### Isolation of exosomes

Exosomes were isolated from 1 ml plasma using the exoRNeasy Serum/Plasma Midi Kit (Qiagen, Germany) and from 300 μl CSF (pool from three rats) using a Total Exosome Isolation (from other body fluids) Kit (Thermo Fisher Scientific, USA) according to the manufacturer's protocol.

### Transmission electron microscopy

Transmission electron microscopy (TEM) was used for the morphological investigation of the exosomes. Briefly, the exosomes were re-suspended in RNase-free water, transferred onto 200-mesh Formvar-coated copper grids, allowed to sit for 1 min, and stained using 1% uranyl acetate for 15 s. After the grids were dried at room temperature, they were observed using a transmission electron microscope (H7650 Hitachi, Japan).

### Nanoparticle-tracking analysis

A NanoSight LM10 Instrument with a blue 405 nm laser by Yezhi Co., LTD. (Shanghai, China) was used to analyse the concentration and size distribution of exosomes. Isolated exosomes were diluted 10 times in particle-free DPBS, and then were injected into the NanoSight sample chamber. The exosomes concentrations and size distributions were assessed by the Nanoparticle Tracking Analysis (NTA) software.

### Western blotting analysis

Exosomal protein was extracted with RIPA buffer (Solarbio, China). Protein concentration was determined according to the BCA method. Approximately 30 ug total protein were separated on SDS-PAGE gels. The blots were blocked and then incubated overnight at 4°C in primary antibodies (anti-CD63, anti-HSP70). After a wash, the membranes were incubated with horseradish peroxidase-conjugated secondary antibody. The transferred proteins were visualized with enhanced chemiluminescence detection kits (Thermo Fisher Scientific, USA).

### RNA isolation

Total RNA from plasma and CSF exosomes was extracted with a miRNeasy® Serum/Plasma kit (Qiagen, Germany) according to the manufacturer's instructions. The RNA was stored at a temperature of −80°C until use. The total RNA was quantified by NanoDrop ND-2000 Spectrophotometer (Thermo Scientific, USA). The RNA isolated from the plasma exosomes was 62–71 and 35–46 ng/ul from CSF exosomes.

### Plasma exosomal miRNA profile

Total RNA extracted from plasma exosomes of the MCAo models and sham controls were reverse transcribed to cDNAs by RT-PCR to produce sRNA libraries, and the libraries were sequenced using an Illumina HiSeq™ 2500 by RiboBio Co., LTD. (Guangzhou, China). The database (miRBase 19.0) contained 765 rat miRNAs in both the sham group and ischaemia groups. The RNA concentration (optical density, 260/280) was similar between each group. Data were corrected for background and were normalized by the mean intensity.

### Detection and quantification of miRNAs by qRT-PCR

The isolated RNA was reverse transcribed using the Mir-X™ miRNA First-Strand Synthesis Kit (TaKaRa, Japan) under the following conditions: 37°C for 60 min and 85°C for 5 s. SYBR Green (TaKaRa, Japan) was used as the fluorescent molecule. A U6 (TaKaRa, Japan) endogenous control was used for normalization, and qRT-PCR was performed using the Roche LightCycler 480 system (Roche Applied Science, Germany). All procedures were performed according to the protocols provided by the manufacturer. Amplification curves, melting curve and cycle threshold (Ct) values were analyzed by the LightCycler 480 Software, and only miRNAs with *Ct* < 35 were included. Data were analyzed using the LightCycler 480 Software 1.5.0 (Roche, Germany). The relative expression of miRNAs was quantified using the 2^−ΔΔCt^ method.

### Statistical analysis

All data were processed using SPSS 22.0. The data are reported as the mean ± SD. One-way analysis of variance followed by the Games-Howell *post-hoc* test was performed for these three experimental groups. Spearman's correlation test was used to analyse correlations between the plasma and CSF of exosomal rno-miR-122-5p and rno-miR-300-3p. Sensitivity, specificity, and accuracy of miRNA were compared using receiver operating characteristic (ROC) analysis. All hypothesis testing was two-tailed, and *p* < 0.05 was considered statistically significant.

## Results

### Assessment of ischaemic injury

**Figure 1:**
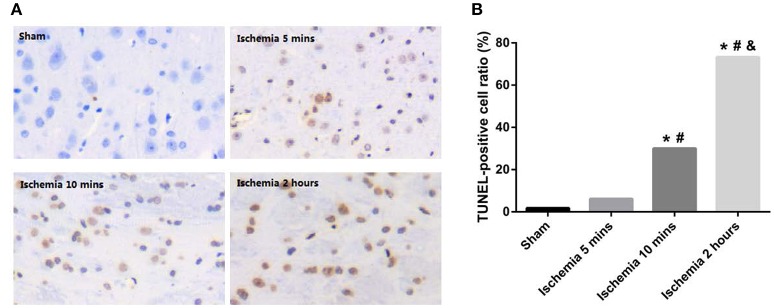
**(A)** Representative histopathologic findings in the frontoparietal cortex are shown by TUNEL staining. **(B)** TUNEL-positive cell ratios of different ischaemia groups (^*^P < 0.001 VS sham; ^#^*P* < 0.001 VS 5 min ischaemia group; ^&^*P* < 0.001 VS 10 min ischaemia group).

**Figure 2:**
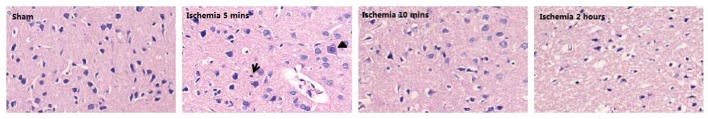
Representative histopathologic findings in the frontoparietal cortex are shown by H&E staining. Selective neuronal necrosis characterized by scattered hypereosinophilic (arrowhead) and shrunken (arrows) neurons.

We evaluated the ischaemic injury of the MCAo based on H&E and TUNEL staining. A few TUNEL-positive cells were present with 5 min of MCAo; ischaemia for 10 min led to an increase in TUNEL-positive cells. After ischaemia for 2 h, the TUNEL-positive cells occupied the major part of the view (Figure 1A). In addition, the total TUNEL-positive ratios were significantly different among the groups, with higher values occurring in the 10 min and 2 h ischaemia groups (*P* < 0.001, Figure 1B). Structural changes of the frontoparietal cortex were observed with H&E staining: selective neuronal necrosis was a consistent feature in the ischaemic frontoparietal cortex starting at 5 min of MCAo; ischaemia for 10 min led to a continued increase of shrunken neurons; ischaemia for 2 h induced necrosis of neurons, with loss of tissue structure and vacuolization of neuropil (Figure 2). The H&E and TUNEL staining results showed that further ischaemic brain damage occurred with prolonged ischaemia.

### Evaluation of isolated exosomes

**Figure 3:**
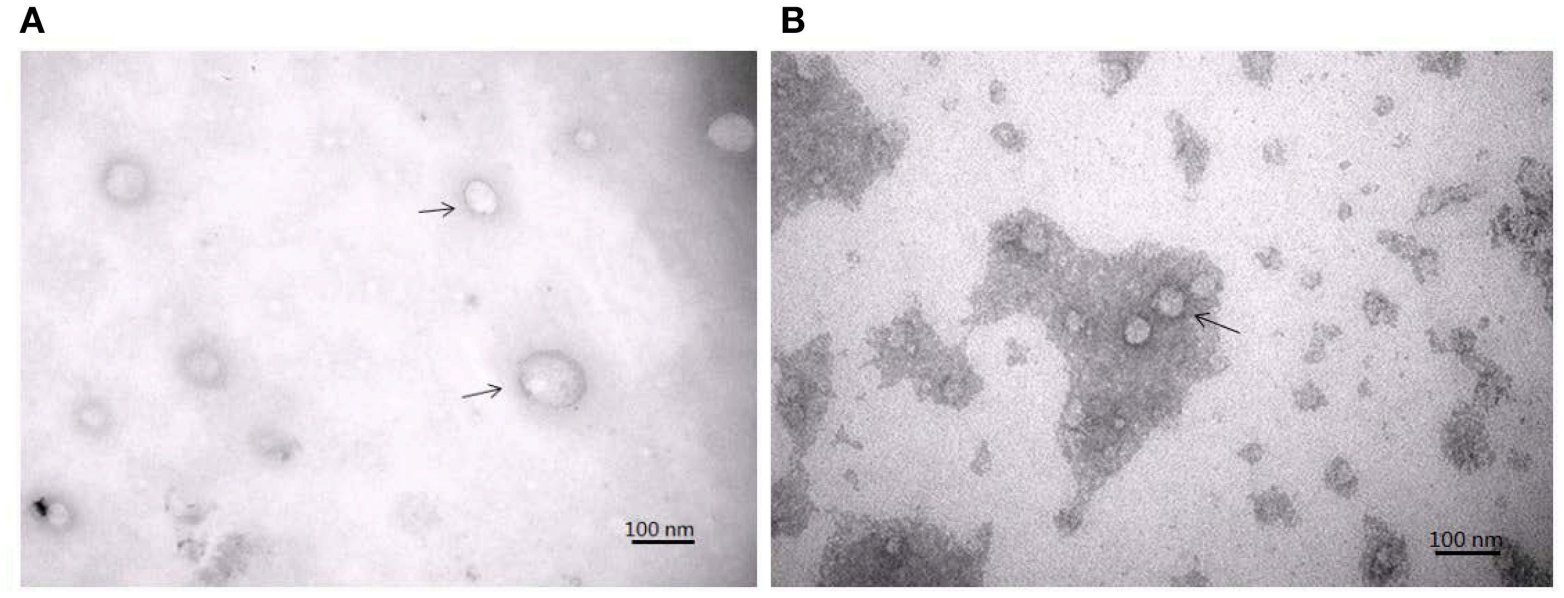
Transmission electron microscopy of isolated plasma exosomes **(A)** and CSF exosomes **(B)**. Exosomes were marked by arrows.

**Figure 4:**
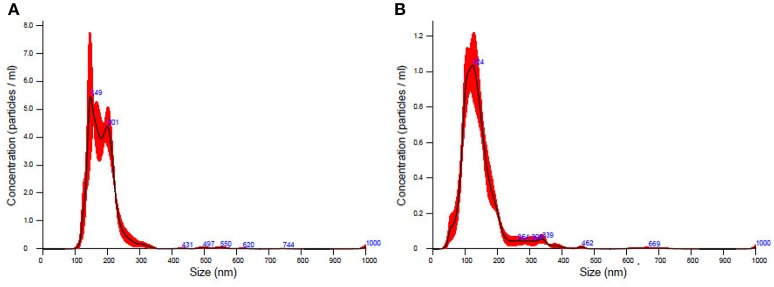
The concentration and size distribution of plasma exosomes **(A)** and CSF exosomes **(B)**.

**Figure 5:**
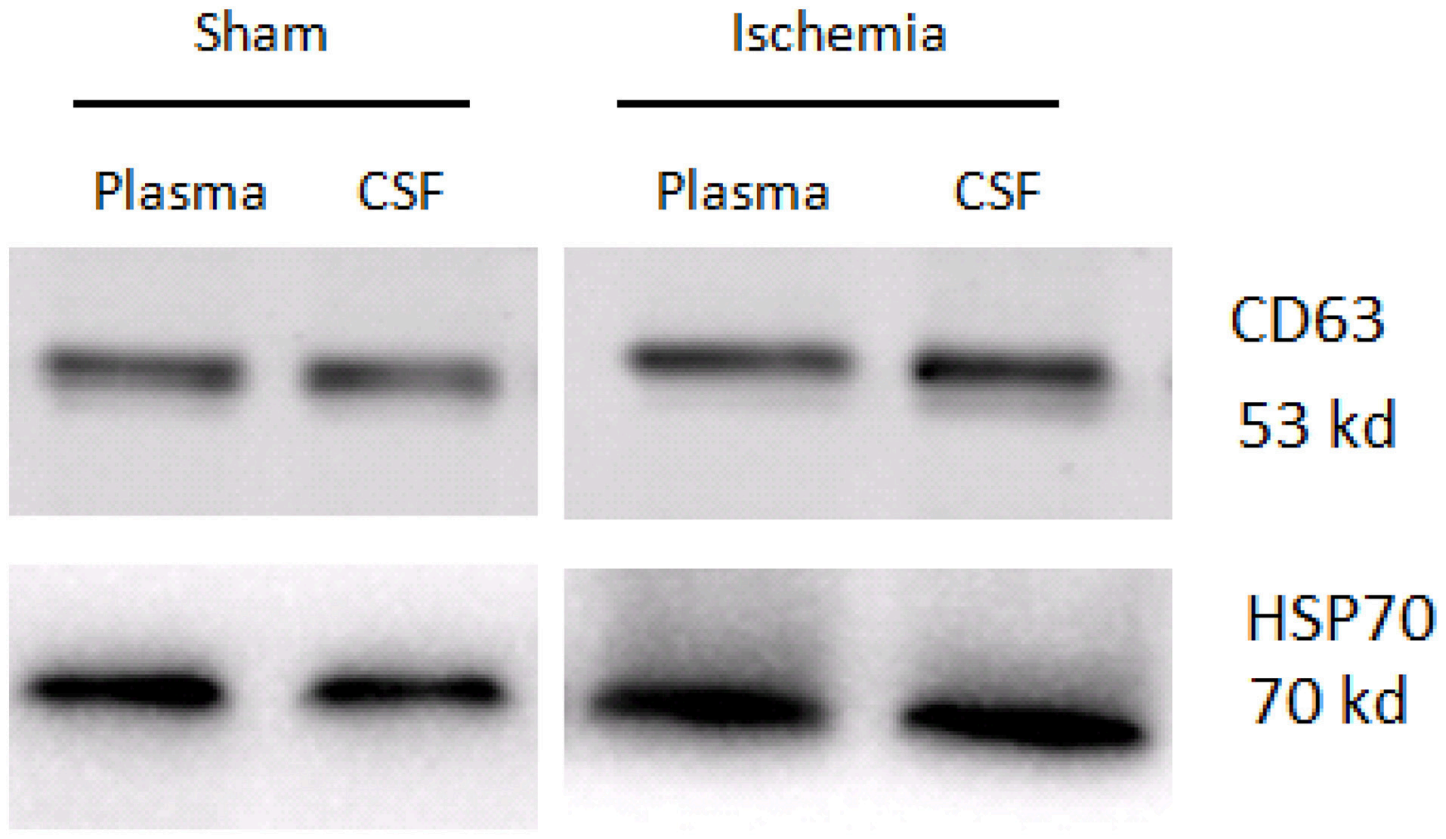
Western blotting analysis of exosome markers, CD63 and HSP70, in the plasma exosomes and CSF exosomes from sham controls and ischaemia groups.

TEM analysis showed a typical size of 30–200 nm diameters in the plasma/CSF exosomal fraction (Figure 3). NanoSight analysis revealed that the mode size of isolated plasma exosomes was 148.1 nm, and the concentration was 4.55e^+008^ ± 1.91e^+007^ particles/ml; the mode size of isolated CSF exosomes was 123.7 nm, and the concentration was 9.62e^+008^ ± 5.09e^+007^ particles/ml (Figure 4). The expression of exosomes markers, HSP70 and CD63, were confirmed by Western blotting (Figure 5). In summary, the exosomes extracted using the exoRNeasy Serum/Plasma Maxi Kit and Total Exosome Isolation (from other body fluids) Kit expressed normal characteristics of exosomes and were suitable for the subsequent experiments.

### Plasma exosomal miRNA profiles

**Figure 6:**
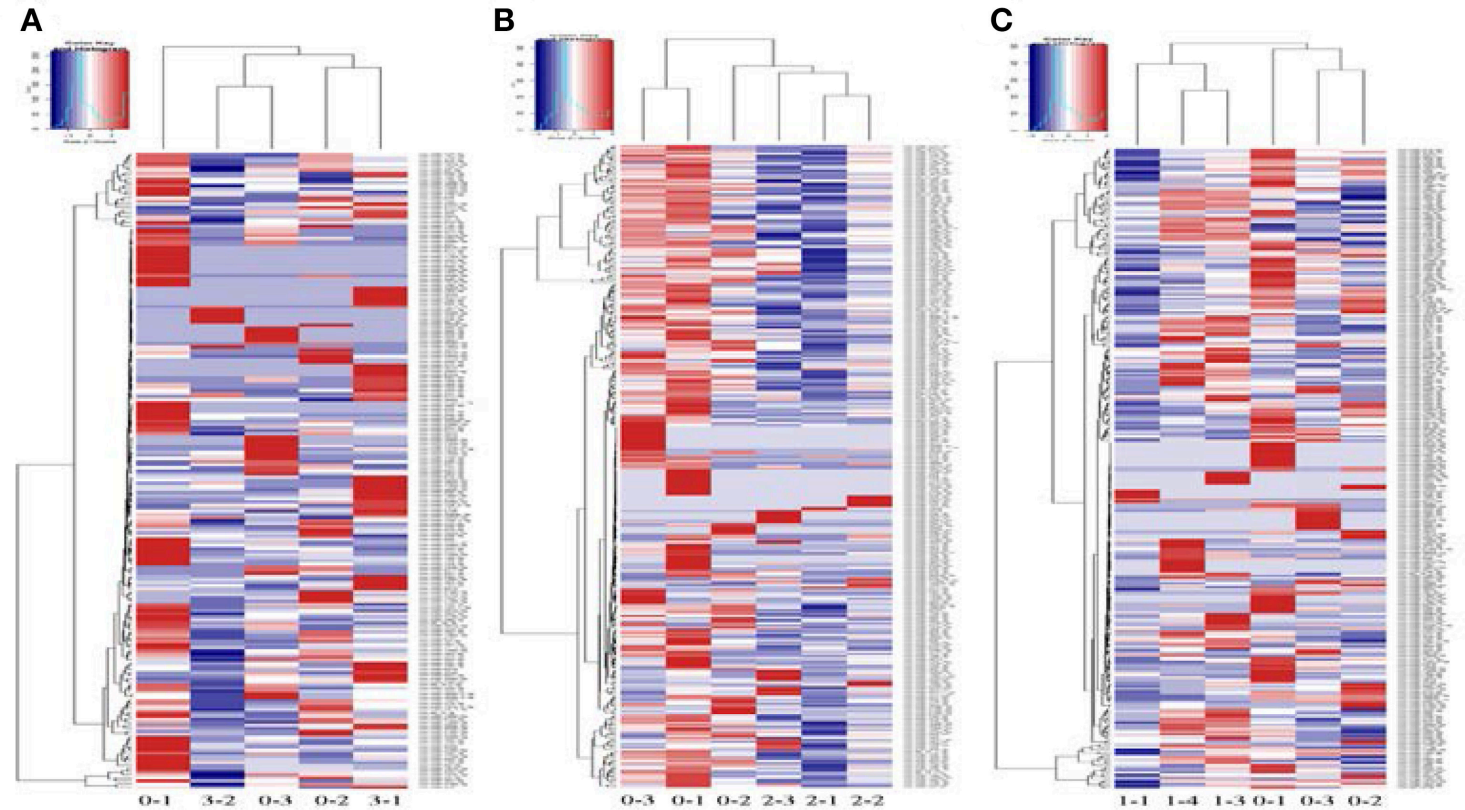
Profile of circulating miRNAs in rat plasma after 5 min (3-1, 3-2) **(A)**, 10 min (2-1, 2-2, 2-3) **(B)** and 2 h (1-1, 1-3, 1-4) **(C)** of focal ischaemia compared with sham controls (0-1, 0-2, 0-3). Heat map diagrams are shown clustering the differentially expressed miRNAs.

High-throughput sequencing was used to identify the expression levels of plasma exosomal miRNAs. (High-throughput sequencing was not used for CSF exosomal miRNAs because the volume of CSF from the rats was too low to meet the requirements.) Expression differences were compared among three rats of the sham group and eight rats of the ischaemia groups (two rats of the 5 min group, three rats of the 10 min group and three rats of the 2 h group). For comparison of differentially expressed miRNAs among multiple samples, we removed the sequences which with < 5 total read counts in the all libraries, the number of reads per million (RPM) clean tags was used to normalize the miRNA abundance of the remaining reads, and we used the edgeR package (Robinson et al., 2010) to confirm significantly differentially expressed miRNAs. Differentially expressed miRNAs were defined as those with a *P* < 0.05 and |fold change| >2, and 7 candidate miRNAs (Table 1) of the sham group VS the 5 min group and 32 candidate miRNAs (Table 2) of the sham group VS the 10 min group were selected and identified via qRT-PCR. The number of specific upregulated and downregulated miRNAs after 2 h of ischaemia (which produced stroke in these studies) was 38 and 25 (data not shown), and the expression profiles of the 2 h ischaemia rats were different than those of the 5 min ischaemia and 10 min ischaemia rats. The Heat map analysis showed the relative miRNA expression levels in ischaemia groups and sham group (Figure 6).

**Table 1:** Plasma miRNAs differentially expressed between sham controls and 5 min ischaemia rats (*P* < 0.05).

**miRNA name**	**Mean expression in sham**	**Mean expression in 5 min ischaemia**	**Fold change**
rno-miR-9a-5p	40.31637	1.912	−4.3982
rno-miR-743b-5p	0.6555	0	−6.0345
rno-miR-871-3p	0.625567	0	−5.9672
rno-miR-196c-3p	0.164533	0.97265	2.5639
rno-miR-3593-3p	0	0.2741	4.7766
rno-miR-130a-5p	0.033733	0.36765	3.4477
rno-miR-200c-3p	19.4618	103.3532	2.4089

**Table 2:** Plasma miRNAs differentially expressed between sham controls and 10 min ischaemia rats (*P* < 0.05).

**miRNA name**	**Mean expression in sham**	**Mean expression in 10 min ischaemia**	**Fold change**
rno-miR-6215	0.451967	4.2442	3.2311
rno-miR-293-5p	2.929333	0.089533	−5.0325
rno-miR-101b-3p	1373.173	119.734	−3.5196
rno-miR-295-3p	1.0644	0	−6.7339
rno-miR-122-5p	90679.6	5146.172	−4.1392
rno-miR-216b-5p	9.258233	0.0947	−6.6112
rno-miR-217-5p	43.29177	1.4398	−4.9102
rno-miR-216a-5p	5.034967	0.125633	−5.3251
rno-miR-196c-3p	0.164533	1.569333	3.254
rno-miR-216a-3p	1.020867	0	−6.6737
rno-miR-499-5p	34.09517	3.526367	−3.2733
rno-miR-216b-3p	1.347933	0	−7.0746
rno-miR-101a-3p	969.4929	119.9824	−3.0144
rno-miR-466b-2-3p	5.3702	0.552267	−3.2815
rno-miR-466b-4-3p	5.3702	0.552267	−3.2815
rno-miR-451-5p	4214.157	408.2909	−3.3676
rno-miR-143-3p	24057.89	3945.56	−2.6082
rno-miR-218a-5p	513.9875	81.62227	−2.6547
rno-miR-329-3p	2.587767	0.114733	−4.4958
rno-miR-410-3p	5.361267	0.432867	−3.6305
rno-miR-130a-5p	0.033733	0.5839	4.1149
rno-miR-376b-5p	3.801333	0.405633	−3.2284
rno-miR-196c-5p	0.029967	0.568067	4.2431
rno-miR-488-3p	12.22793	54.08377	2.145
rno-miR-708-3p	22.70093	3.601767	−2.656
rno-miR-300-3p	17.12423	1.9431	−3.1396
rno-miR-32-5p	22.63673	2.3557	−3.2644
rno-miR-301a-3p	1.930567	0.1544	−3.6443
rno-miR-881-3p	0.3811	0	−5.2521
rno-miR-1-3p	22405.97	2474.263	−3.1788
rno-miR-203b-3p	20.08503	2.945433	−2.7696
rno-miR-3068-5p	0.199633	1.0601	2.409

### Detection of plasma/CSF exosomal miRNAs

First, we investigated the expression of plasma exosomal miRNAs in sham subjects and ischaemia subjects (data not shown). Then, to test whether plasma exosomal miRNAs could function as potential biomarkers, we analyzed them in CSF exosomes to demonstrate that peripheral changes reflect modifications occurring in the brain. Finally, we identified two miRNAs (rno-miR-122-5p and rno-miR-300-3p) that had common expression trends in CSF and plasma exosomes and had significant differences between the sham group and ischaemia group (5 or 10 min).

### Expression levels of exosomal rno-miR-122-5p in plasma and CSF

**Figure 7:**
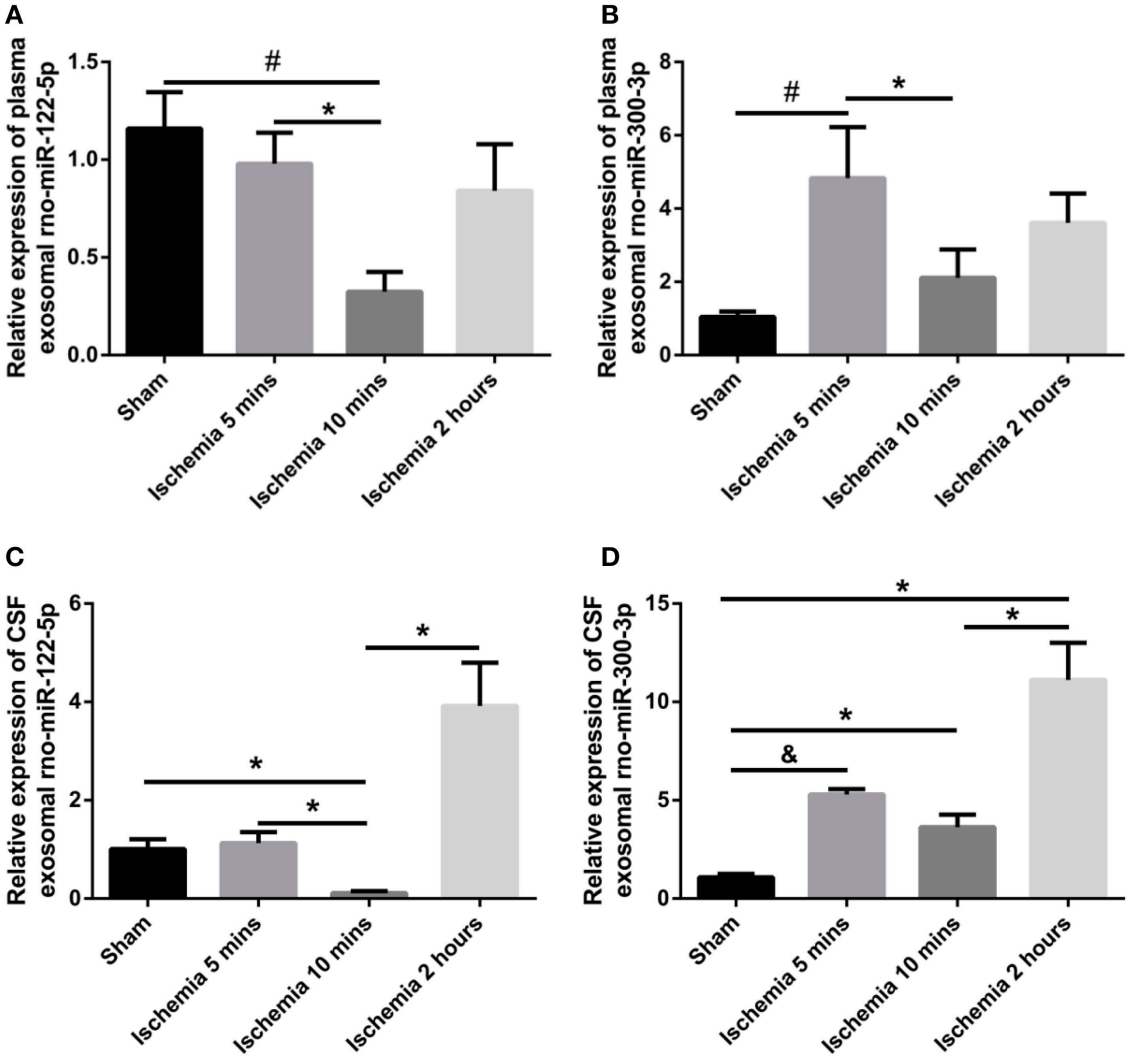
Expression levels of plasma/CSF exosomal rno-miR-122-5p and rno-miR-300-3p in sham controls and the ischaemia groups (*n* = 5 each group; ^*^P < 0.05, ^#^*P* < 0.01, ^&^*P* < 0.001). **(A,B)** Relative expression of plasma exosomal rno-miR-122-5p and rno-miR-300-3p. **(C,D)** Relative expression of CSF exosomal rno-miR-122-5p and rno-miR-300-3p.

**Figure 8:**
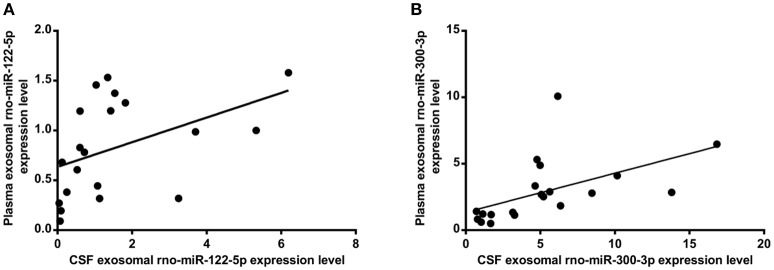
Correlation between the plasma and CSF of levels of exosomal rno-miR-122-5p **(A)** (*R* = 0.632, *P* < 0.005), rno-miR-300-3p **(B)** (*R* = 0.719, *P* < 0.001).

The exosomal rno-miR-122-5p levels were significantly downregulated in the plasma of 10 min ischaemia rats compared with the sham controls and 5 min ischaemia rats (*P* < 0.01, *P* < 0.05, respectively, Figure 7A). Significant correlations were found between the plasma and CSF levels of exosomal rno-miR-122-5p via correlation analysis (*R* = 0.632, *P* < 0.005, Figure 8A), but compared with the 10 min ischaemia rats, the 2 h ischaemia rats had higher levels of exosomal rno-miR-122-5p in the CSF (*P* < 0.05, Figure 7C).

### Expression levels of exosomal rno-miR-300-3p in plasma and CSF

The exosomal rno-miR-300-3p levels were significantly upregulated in the plasma of 5 min ischaemia rats compared with the sham controls and 10 min ischaemia rats (*P* < 0.01 and *P* < 0.05, respectively, Figure 7B). Significant correlations were found between the plasma and CSF levels of exosomal rno-miR-300-3p by correlation analysis (*R* = 0.719, *P* < 0.001, Figure 8B), but compared with the sham controls and 10 min ischaemia rats, the 2 h ischaemia rats had higher levels of exosomal rno-miR-300-3p in the CSF (*P* < 0.05, Figure 7D).

**Figure 9:**
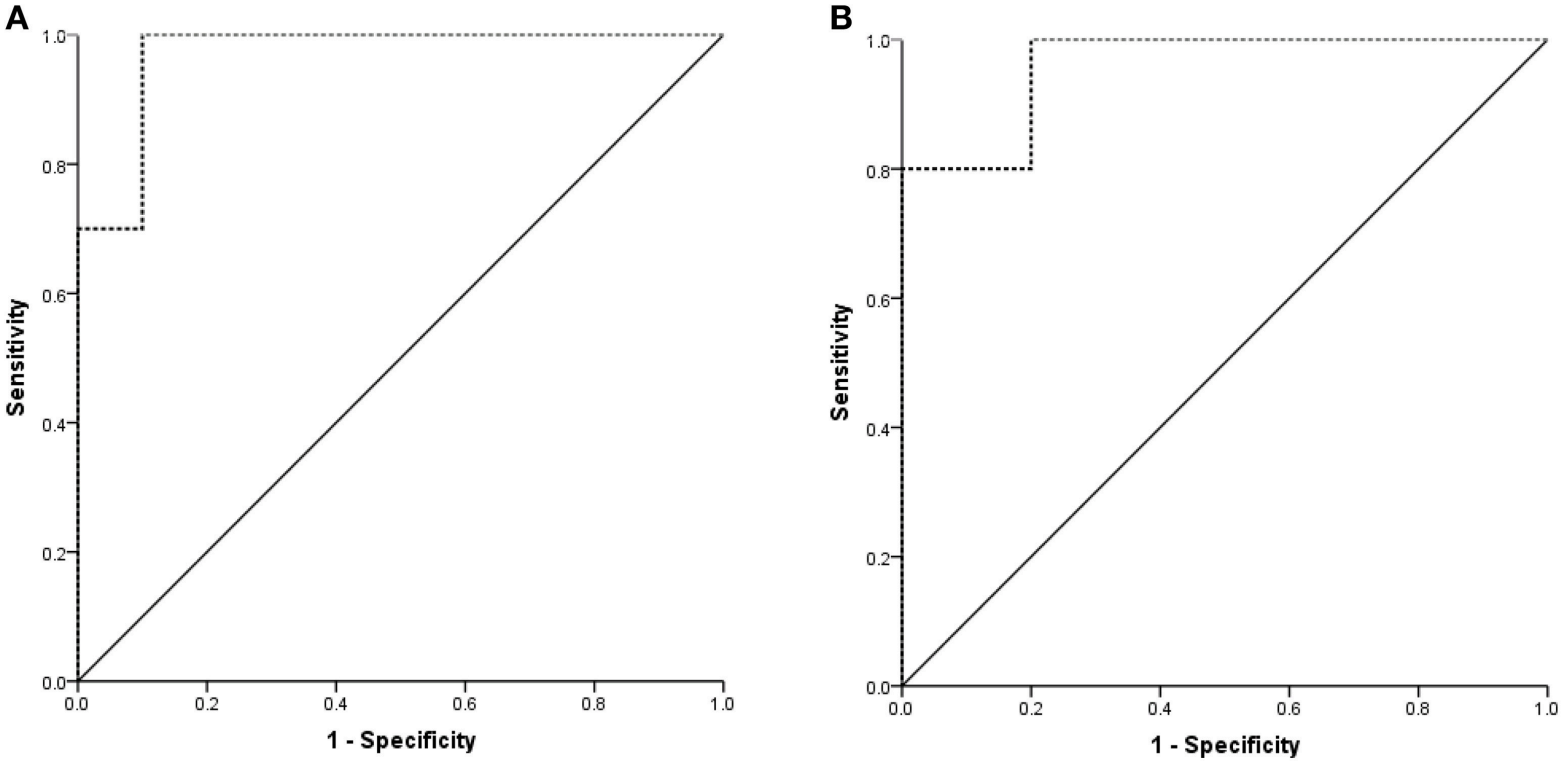
Diagnostic value of plasma exosomal rno-miR-122-5p and rno-miR-300-3p in rats. **(A)** Sham controls VS 5 min ischaemia group; the AUC of plasma exosomal rno-miR-300-3p was 0.970 (95%CI: 0.902-1.000). **(B)** Sham controls VS 10 min ischaemia group; the AUC of plasma exosomal rno-miR-122-5p was 0.960 (95%CI: 0.843-1.000).

Next, we generated ROC curves to evaluate the potential value of plasma exosomal rno-miR-300-3p and rno-miR-122-5p as biomarkers for the diagnosis of TIA for different times (5 min and 10 min ischaemia). When the expression in the 5 min ischaemia group was compared to that in the sham controls, the AUC for the plasma exosomal rno-miR-300-3p was 0.970 (95%CI: 0.902-1.000, Figure 9A). When the expression in the 10 min ischaemia group was compared to that in the sham controls, the AUC for the plasma exosomal rno-miR-122-5p was 0.960 (95%CI: 0.843-1.000, Figure 9B). These findings suggested that plasma exosomal rno-miR-300-3p and rno-miR-122-5p are very valuable for diagnosing TIA in rats.

## Discussion

TIA is defined as a type of cerebrovascular disease which are usually associated with a focal neurological deficit and does not result in permanent damage of brain tissue (Whisnant et al., 1990). TIA was considered to be one of the major risk factors for IS, and eventually to IS (Streifler et al., 1995; Johnston et al., 2000). As reported in previous study, TIA-induced tissue changes may vary from an ischaemia-tolerant state (Johnston, 2004) to clinically silent infarction. In addition, repeated TIAs may be related with cognitive decline and brain atrophy (Bakker et al., 2003; Walters et al., 2003), even in “MRI-negative” TIA patients; this indicates as the limits of MRI, some degree of permanent brain injury of TIA were not detected. Our study examined circulating biomarkers for TIA in rats and might provide a theoretical foundation for further research in humans.

Plasma or serum miRNAs have been identified as diagnostic biomarkers for disease (Fichtlscherer et al., 2010; Zampetaki et al., 2010; Huang et al., 2014); however, due to the low concentrations of plasma and serum miRNAs, their use as biomarkers is limited (Huang et al., 2016). Surprisingly, exosomes transport miRNAs and show miRNA enrichment. Studies have reported that within a few minutes after ischaemia onset, various brain functions begin to breakdown as a direct consequence of ischaemia, including changes in gene expression (Hossmann, 2006; Christensen, 2007); the brain is a major source of CSF exosomal miRNAs, which may reflect brain pathophysiology (Yagi et al., 2016). More importantly, exosomes may play an important role as carriers of miRNAs through the BBB into the circulatory system (Cheng et al., 2013). In addition, plasma exosomal miRNAs are not as easily degraded and are more abundant than those found directly in plasma and serum, confirming that exosomal miRNAs are optimal biomarkers for disease.

In this study, we occluded the middle cerebral artery of rats for 5 and 10 min to establish the TIA model and evaluated the histopathologic change via H&E and TUNEL staining. The results showed minute ischaemic changes after 5-min MCAo, and the longer the MCAo duration, the more abundant were these changes (Figures 1, 2). These findings are similar to those from a previous report (Pedrono et al., 2010). MiRNAs are essential molecules in the process of intracellular modulation for gene expression, and the types of miRNAs and their levels are emerging as biomarkers for various pathological conditions (Waldman and Terzic, 2008). In our study, 2 miRNAs (rno-miR-122-5p and rno-miR-300-3p) were chosen from 39 candidate miRNAs that had common expression trends in CSF and plasma exosomes. We analyzed the expression levels of plasma exosomal rno-miR-122-5p and rno-miR-300-3p in rats after episodes of brief focal cerebral ischaemia (5 and 10 min) compared with IS (2 h) and sham controls. We found that the expression levels of exosomal rno-miR-122-5p were significantly downregulated in the plasma and CSF of 10 min ischaemia rats compared with the sham controls and 5 min ischaemia rats. Exosomal rno-miR-300-3p levels in the plasma and CSF of 5 min ischaemia rats were significantly upregulated compared to those of the sham controls and 10 min ischaemia rats. Although we found that the expression levels of exosomal rno-miR-122-5p and rno-miR-300-3p were significantly upregulated in the CSF of the 2 h ischaemia rats compared with the 10 min ischaemia rats, there were no significant differences in these exosomal miRNAs in the plasma, even though they showed an increasing trend. These results indicate that the expression differences of these exosomal miRNAs are greater in the CSF than the plasma. We hypothesized that the plasma rno-miR-300-3p and rno-miR-122-5p, which were derived from CSF, were reduced by the BBB (Sørensen et al., 2014). All the above results suggest that plasma exosomal rno-miR-122-5p and rno-miR-300-3p are promising biomarkers for diagnosing TIA in rats. The diagnostic performance of these miRNAs in rats was measured by ROC analysis, which indicated that rno-miR-300-3p is useful for distinguishing those in the 5 min TIA group from sham controls (AUC was 0.970), and rno-miR-122-5p can distinguish those in the 10 min TIA group from sham controls (AUC was 0.960). Based on the experimental data and analysis results, we can draw the following conclusion: compared with the expression in sham controls, the expression level of plasma exosomal rno-miR-122-5p was significantly decreased and there was no significant difference in the level of rno-miR-300-3p, or rno-miR-300-3p was significantly increased and there was no significant difference in the level of rno-miR-122-5p, we could speculate it is in the TIA; if both the rno-miR-122-5p and rno-miR-300-3p showed no significant difference, then, we could speculate it's not TIA. To our knowledge, this is the first report to evaluate the utility of plasma exosomal miR-300-3p and miR-122-5p as biomarkers of TIA in the rat.

Recent evidence showed that miR-122-5p plays important roles in various human diseases. Previous reports have indicated that the miR-122-5p/miR-133b ratio could as a specific early prognostic biomarker for acute myocardial infarction (Cortez-Dias et al., 2016), and miR-122-5p upregulation can trigger the compensatory response of LPIN1 and CTDNEP1 in hepatosteatosis (Naderi et al., 2017). In addition, miR-122-5p plays a role in the pathophysiology of heart failure (Marques et al., 2016). However, there is no information available regarding miR-122-5p in TIA. Stroke and TIA are considered clinical manifestations of atherosclerotic disease due to sustaining vascular inflammation and finally atherothrombosis of the carotid arteries (Markus et al., 2016). Many pathophysiological mechanisms, including excitotoxicity, calcium dysregulation, oxidative stress, apoptosis and inflammation, contribute to cerebral infarcts (Dirnagl et al., 1999). Emerging evidence has indicated that miRNAs are involved in immune response (Xiao and Rajewsky, 2009; O'Connell et al., 2010; Njock et al., 2015) and the response to cerebral ischaemia (Ouyang et al., 2013; Wen et al., 2015). Among various miRNAs, miR-122-5p is also an inflammation-related miRNA that plays an important role in the pathogenesis of dyslipidaemia and apoptosis (Hromadnikova et al., 2015; Liu et al., 2016) and is positively correlated with the regulation of NFκB and inflammatory activity (Matsuura et al., 2016; Weaver et al., 2016). Few studies have investigated miR-300-3p, but several animal experiments suggested that the miR-300-3p is associated with the puromycin aminonucleoside nephropathy (Sun et al., 2013) and may play an important role in respiration and movement in the sea cucumber (Wang et al., 2015). We hypothesized that miR-300-3p may be a target for prevention and treatment of TIA. In summary, we demonstrated that both miR-122-5p and miR-300-3p are related to cerebral ischaemic injury. Further studies and analysis of miR-300-3p and miR-122-5p are needed.

In conclusion, the differential expression of plasma exosomal miR-300-3p and miR-122-5p suggests that these two plasma exosomal miRNAs can be used as diagnostic markers for TIA in rat and as potential therapeutic targets.

## Author contributions

All authors listed have made a substantial, direct and intellectual contribution to the work, and approved it for publication.

### Conflict of interest statement

The authors declare that the research was conducted in the absence of any commercial or financial relationships that could be construed as a potential conflict of interest.
